# Brazilian version of the CHOP INTEND scale: cross-cultural adaptation and validation

**DOI:** 10.1055/s-0043-1772832

**Published:** 2023-10-04

**Authors:** Renalli Manuella Rodrigues Alves, Alessandra Paula de Melo Calado, Vanessa Van Der Linden, Maria Aparecida Ferreira Chaves Bello, Lívia Barboza de Andrade

**Affiliations:** 1 Instituto de Medicina Integral Professor Fernando Figueira, Pós-graduação em Saúde Integral, Recife PE, Brazil.; 2 Hospital Otávio de Freitas, Departamento de Reabilitação, Recife PE, Brazil.; 3 Hospital Maria Lucinda/Rarus, Serviço de Doenças Raras, Recife PE, Brazil.; 4 Universidade Federal de Pernambuco, Centro de Ciências da Saúde, Recife PE, Brazil.; 5 Associação de Assistência à Criança Deficiente, Serviço de Fisioterapia, Recife PE, Brazil.

**Keywords:** Muscular Atrophy, Spinal, Growth and Development, Validation Study, Atrofia Muscular Espinal, Crescimento e Desenvolvimento, Estudos de Validação

## Abstract

**Background** Spinal muscular atrophy (SMA) is a rare genetic disease that causes progressive muscle weakness and impacts motor function. The type I is the most severe presentation and affects infants before 6 months old. In addition, the instruments available for assessing motor function have limitations when applied to infants with neuromuscular diseases and significant muscle weakness.

**Objective** To translate, cross-culturally adapt, and validate the Children's Hospital of Philadelphia Infant Test of Neuromuscular Disorders (CHOP INTEND) to Brazilian Portuguese.

**Methods** The present study comprised the translation, synthesis of translations, backtranslation, consolidation by a committee of experts, and test of the final version of the CHOP INTEND in 13 patients with SMA type I. We also assessed the content validity and reliability of the translated version.

**Results** The scale was translated considering semantic, structural, idiomatic, and cultural aspects. All agreement rates were > 0.8, the overall content validity index of the instrument was 0.98, and inter-rater reliability using the intraclass correlation coefficient was 0.998.

**Conclusion** The Brazilian version of the CHOP INTEND met semantic and technical equivalence criteria with the original version and was valid and reliable for patients with SMA type I.

## Resumo

**Antecedentes** A atrofia muscular espinhal (AME) é uma doença genética rara que provoca fraqueza muscular progressiva com impacto sobre a motricidade dos pacientes. A AME tipo I é considerada o tipo mais grave e acomete lactentes antes dos 6 meses de idade. As escalas disponíveis para avaliação das aquisições motoras mostram limitações para uso com crianças pequenas com doenças neuromusculares e fraqueza importante.

**Objetivo** Realizar a tradução, adaptação transcultural e validação para a língua portuguesa do Brasil da Children's Hospital of Philadelphia Infant Test of Neuromuscular Disorders (CHOP INTEND, na sigla em inglês).

**Métodos** O presente estudo seguiu as etapas de tradução, síntese das traduções, retrotradução, consolidação por comitê de especialistas e teste com 13 pacientes com AME tipo 1. Foi avaliada a validade de conteúdo e a confiabilidade do instrumento.

**Resultados** A escala foi traduzida considerando os aspectos semânticos, estruturais, idiomáticos e culturais. Todas as taxas de concordância foram > 0,8. O índice de validade de conteúdo geral do instrumento foi de 0,98. A confiabilidade interavaliadores analisada através do coeficiente de correlação intraclasse (ICC, na sigla em inglês) demonstrou um valor de ICC = 0,998.

**Conclusão** A versão da CHOP INTEND em português atende aos critérios de equivalência semântica e técnica em relação à versão original e apresenta validade de conteúdo e confiabilidade para seu uso na população de pacientes com AME tipo I.

## INTRODUCTION

The discovery of the molecular and genetic bases of spinal muscular atrophy (SMA) linked to chromosome 5q stimulated the search for new clinical treatments and sensitive markers to monitor the evolution of patients. 1 2 3 4 In the current scenario, disease-modifying therapies associated with multidisciplinary care showed promising results in clinical trials and real-life studies. 5 6 7

Spinal muscular atrophy is a rare genetic disease that affects the motor neurons, causing hypotonia, progressive muscle weakness, and delay and loss of motor function. 8 9 The disease is classified according to age of onset of symptoms and maximum motor function achieved, which results in a wide spectrum of phenotypes grouped into four types (I, II, III, and IV). 1 8 9

About 70% of SMA cases are classified as the most severe type (I), characterized by early symptom onset (that is, before 6 months of age), difficulty in cervical control, and inability to sit and roll over. 8 10 Over time, the loss of motor function results in reduced movements and intolerance to some postures. 3 8 In addition, the progression of the disease leads to respiratory impairments, paradoxical breathing, chest restriction, bell-shaped chest, hypoventilation, and respiratory failure. 9 11 12

The lack of standardized instruments to assess the motor behavior of infants with limited motor function and clinical frailty encouraged the development of the Children's Hospital of Philadelphia Infant Test of Neuromuscular Disorders (CHOP INTEND), which may also be applied as outcome in clinical trials. 13 14 15 The scale presents good sensitivity, reliability, and responsiveness over time and may be an ideal instrument for assessing infants with SMA type I who cannot sit. 13 14

The CHOP INTEND was based on the Test of Infant Motor Performance with the addition of new created items to analyze the spontaneous activity, directed movements, and reflexes of patients. The 16 items of the scale can be quickly applied and describe nongravity and counter-gravity movements organized from the simplest to the most complex; scores are graded from 0 (worst function) to 4 (best response). 13 14 15 16

The Hammersmith Infant Neurological Examination and Motor Function Measure are two instruments translated into Brazilian Portuguese and applied to patients with neuromuscular diseases. However, these instruments may not present adequate sensitivity to patients with SMA type I with limited movements and unable to sit. 17 18

Thus, our study aimed to translate and cross-culturally adapt the CHOP INTEND scale into Brazilian Portuguese and assess its content validity and reliability.

## METHODS

**Figure 1:**
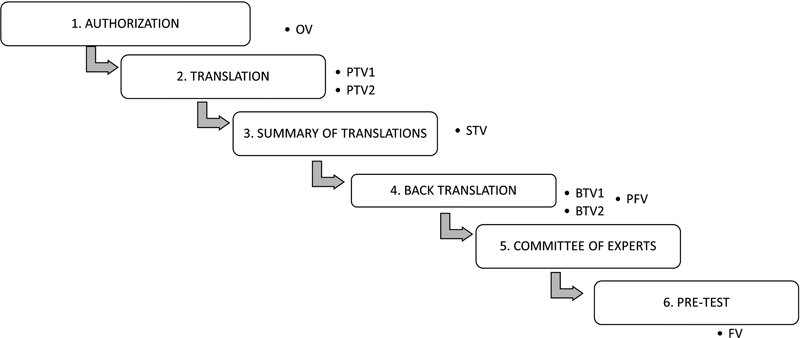
Stages of the transcultural adaptation of the CHOP INTEND to Brazilian Portuguese. Abbreviations: OV, original version; PTV1 and PTV2, Portuguese translated version; STV, synthesis of the translated versions; BTV1 and BTV2, backtranslation to English; PFV, prefinal version; TV, test version; FV, final version.

The translation and cross-cultural adaptation of the CHOP INTEND scale was conducted according to Reichenheim et al. and the Consensus-Based Standards for the Selection of Health Measurements Instruments. 19 20 21 The study was approved by the research ethics committee of the Instituto de Medicina Integral Prof. Fernando Figueira (no. 2.644.780). Besides, legal guardians of patients signed the informed assent form, and experts involved in the research signed the informed consent form. The study was conducted according to the stages shown in Figure 1 .

In the authorization stage, permission to conduct the cross-cultural adaptation was requested by e-mail and accepted by the author of the original article. For the direct translation, two independent and qualified Brazilian bilingual translators (one healthcare professional) translated the CHOP INTEND scale into Brazilian Portuguese. This process resulted in two translated versions (Portuguese translated version 1 [PTV1] and Portuguese translated version 2 [PTV2]).

In the synthesis of the translation, a meeting was held with the translators and researchers to evaluate linguistic and contextual differences and obtain a single version in Brazilian Portuguese. Versions were compared, differences were identified, and adaptations were made until reaching a consensus on the synthesis of the translated versions (STV).

For the backtranslation stage, the STV was backtranslated into English by two independent English-speaking bilingual translators (one healthcare professional), resulting in two backtranslated versions (backtranslated version 1 [BTV1] and backtranslated version 2 [BTV2]). Afterward, researchers compared the two versions and discussed and adjusted possible semantic and conceptual differences that could compromise the meaning of words. Finally, the instrument was translated into Brazilian Portuguese to generate a prefinal version (PFV).

In the next stage, a face-to-face committee of 10 experts with practical experience in pediatric neurology (1 neurologist and 9 physical therapists) was formed; 6 had a Master's degree, and 2 had a PhD.

The original version, PTV1, PTV2, STV, BTV 1, BTV 2, and PFV were analyzed. The committee analyzed the equivalence between the translated and original instruments considering four aspects: semantic equivalence, which assessed grammatical and vocabulary issues and analyzed whether words had the same or more than one meaning; idiomatic equivalence, which observed whether the cultural meaning between languages was maintained in the translated items; experiential equivalence, which analyzed whether a given sentence or word was applicable in the target culture; and conceptual equivalence, which assessed whether a given term or expression had the same meaning across different cultures, even if properly translated. 22 23

The committee also discussed whether terms were suitable for the pediatric population and could be applied to different regions of the country; they also added or replaced inappropriate, irrelevant, or ambiguous items with other suitable terms. Therefore, the committee helped elaborate the PFV used in the tests.

The content validity evaluated whether each item of the translated version of the CHOP INTEND was able to measure the motor function of patients with SMA type I. A 4-point Likert scale was applied with the following considerations: (1) the item is not relevant or not clear, (2) the item needs major revision to be relevant or clear, (3) the item is relevant or clear and needs minor revision, and (4) the item is relevant or clear.

The content validity index (CVI) used the following calculation to assess the agreement for each item between experts: CVI = total number of responses 3 or 4 / total number of responses. A CVI > 0.7 was considered acceptable. 22 24

In the pretest stage, the CHOP INTEND scale adapted to Brazilian Portuguese was applied to 13 patients with SMA type I treated at a referral rehabilitation service for neuromuscular diseases in the state of Pernambuco, Brazil. Inclusion criteria were patients aged from zero to 8 years old with a diagnosis of SMA 5q type I (genetic test), under noninvasive mechanical ventilation, and clinically stable during the assessment. We excluded those agitated or crying (or both); with confirmed cognitive, visual, or hearing deficits that hindered the test; or unable to maintain spontaneous breathing without noninvasive mechanical ventilation during the assessment.

The pretest assessed the quality, feasibility, and applicability of the translation and cross-cultural adaptation to ensure the understanding and clarity of the instrument and verify whether the time spent filling it was convenient. Initially, a single researcher assessed each patient using the translated version of the CHOP INTEND. The test was filmed and exhibited to three researchers who independently considered the highest score for each item on the scale. This strategy was used to avoid multiple assessments in a short period since patients with SMA type I are more vulnerable and less resistant to manipulation. The same precaution was used during the development of the original version of the CHOP INTEND scale. 13 14

### Statistical analysis

IBM Statistics for Windows version 20.0 (IBM Corp., Armonk, NY, USA) was used for data analysis. Descriptive statistics characterized the patients, and the intraclass correlation coefficient (ICC) 22 25 assessed the inter-rater agreement. Reliability was classified as unacceptable (< 0.70), acceptable (between 0.71 and 0.79), very good (between 0.80 and 0.89), or excellent (> 0.90). 26

## RESULTS

The researchers synthesized the translation based on PTV1 and PTV2. Acronyms and abbreviations were replaced with full terms to adequate the terms according to clinical and patient identification data. The term *Gtube* was replaced by *gastrostomia* , *BiPAP* by v *entilação não-invasiva* , *upper respiratory infection (URI)* by *infecção respiratória* , *medical register (MR)* by *registro* , *date of evaluation (DOE)* by *data de avaliação* , *date of birth (DOB)* by *data de nascimento* , and *hours (HRS)* by *horas* .

The researchers also discussed the best term to apply in the case of word divergence between the translators. The consensus applied by researchers for developing the STV is shown in Table 1 .

**Table 1:** Stage 1 of the cross-cultural adaptation process

Item	Original version	PTV1	PTV2	STV
1, 2	Observe throughout testing	Observe durante o teste	Observar através do teste	Observe durante o teste
1	Antigravity shoulder movement	Movimento do ombro antigravidade	Movimento antigravitacional do ombro	Movimento antigravitacional do ombro
1	Achieves elbow off surface	Alcança cotovelo sem apoio fora da superfície	Consegue retirar o cotovelo da superfície	Consegue retirar o cotovelo da superfície
1	Achieves hand and forearm off surface	Alcança mão e antebraço sem apoio em superfície	Consegue retirar a mão e o antebraço da superfície	Consegue retirar a mão e o antebraço da superfície
2	Antigravity hip movement	Movimento do quadril antigravidade	Movimento antigravitacional do quadril	Movimento antigravitacional do quadril
2	Achieves feet and knees off surface	Alcança pés e joelhos sem apoio em superfície	Consegue retirar os joelhos e os pés da superfície	Consegue retirar os joelhos e os pés da superfície
2	Antigravity hip adduction/internal rotation	Movimento do quadril adução/rotação interna antigravidade	Movimento antigravitacional de adução e rotação interna	Movimento antigravitacional de adução e rotação interna do quadril
2	Knees off surface	Joelhos sem apoio em superfície	Joelhos fora da superfície	Joelhos fora da superfície
2	Active gravity eliminated knee movement	Movimento ativo do joelho com gravidade eliminada	Movimento do joelho sem ação da gravidade	Movimento ativo do joelho sem ação da gravidade
3	Hand grip	Força do aperto de mão	Garra da mão	Força do aperto de mão
3	Grip strength: place finger in palm and lift until shoulder comes off surface observe when infant loses grasp	Observar quando a criança perde a aderência.	Observe quando a criança começa a perder força na garra.	Observe quando a criança começa a perder a força de preensão manual.
4	Turns head part way back to midline	Gira a cabeça parte da volta para a linha média	Vira a cabeça até metade do caminho para a linha média	Vira a cabeça até parte do caminho de volta para a linha média
6	Elicited from	Iniciado com	Observado a partir de	Iniciado a partir de
6	To allow infant to attempt to derotate body	Para permitir que a criança tente derrotar o corpo	Para permitir que a criança tente sair da rotação do corpo	Para permitir que a criança tente rodar o corpo
6, 7	Roll away from the side tested	Rolar para o outro lado	Rolar para longe do lado testado	Rolar para longe do lado testado
7	Allow infant to derotate	Permitindo que a criança gire.	Permite a criança a sair da rotação	Permite a criança a sair da rotação
7	Head righting	Alinhamento lateral da cabeça	Endireitamento lateral da cabeça	Endireitamento lateral da cabeça
8	Restrain lower arm if needed	Mantenha o braço para baixo se necessário	Conter braço para baixo se necessário	Conter o braço inferior se necessário
8	Prompt reach for a toy presented at arm's length at shoulder level	Alcança um brinquedo levando os braços na altura do nível dos ombros	Imediatamente tenta alcançar um brinquedo no comprimento dos braços no nível do ombro	Alcança prontamente um brinquedo apresentado no comprimento do braço no nível do ombro.
8	Clears hand from surface with antigravity arm movement	Tira a mão da superfície com o braço se movimentando anti-gravidadeie	Libera a mão da superfície com movimento antigravitacional do braço	Libera a mão da superfície com movimento antigravitacional do braço
9	Shoulder flexion and elbow flexion	Ombro flexionado e cotovelo flexionado	Flexão do ombro e flexão do cotovelo	Flexão do ombro e flexão do cotovelo
10	Sitting in lap or over edge of mat with head and trunk support 20° recline	Sentado no colo ou no tapete com a cabeça e o suporte do troco com 20° reclinado	Senta no colo ou na borda do tablado com suporte na cabeça e no tronco 20 graus de reclíneo	Sentado no colo ou na borda do tablado com suporte na cabeça e no tronco reclinado em 20 graus
10	Tickle plantar surface of foot	Uma superfície que gere cócegas leves nos pés	Fazer cosquinha na superfície plantar do pé ou gentilmente belisca o dedo do pé	Fazer cócegas na superfície plantar do pé
11	Facing outward	Enfrentando para fora	Olhando para frente	Virado para a frente
11	Stroke the foot or pinch the toe	Acaricie ou belisque o dedo do pé	Toca o pé ou belisca o dedo do pé	Tocar o pé ou beliscar o dedo do pé
12	Place the infant in ring sit with head erect and assistance given at the shoulders (front and back).	Coloque o bebê num carregador, sente-se com a cabeça ereta e apoiada nos ombros (frente e costas).	Colocar a criança sentada em anel com a cabeça ereta e dando assistência nos ombros (na frente e atrás).	Colocar a criança sentada em anel com a cabeça ereta e dar assistência nos ombros (na frente e atrás).
12	May delay scoring a grade of 1 and 4 until end of test	Pode atrasar numa escala 1 e 4 até fim do teste	Pode atrasar numa escala 1 e 4 até fim do teste	Pode haver um atraso no escore dos graus 1 e 4 até o final do teste
12	Attains head upright from flexion and turns head side to side	Realiza a flexão da cabeça e dá voltas com a cabeça de um lado para o outro	Alcança a cabeça na vertical partindo da flexão e gira a cabeça de um lado para outro	Alcança a cabeça na vertical partindo da flexão e gira a cabeça de um lado para outro
12	Bobbing head control	Dificuldade para controlar da cabeça	Controle instável com a cabeça balançando	Controle instável com a cabeça balançando
12	Head hangs	A cabeça trava	A cabeça fica pendurada	A cabeça fica pendurada
13.14	Score with item	Junto com a pontuação do item	Fazer o escore com o item	Fazer o escore com o item
15.16	Held in one hand upper abdomen	Com uma mão parada no abdômen.	Mantido por uma mão no abdômen superior	Mantido por uma mão no abdômen superior
15	Stroke along spine from neck to sacrum	Leve a cabeça ao longo da coluna, desde o pescoço ao sacro	Leve a cabeça ao longo da coluna, desde o pescoço ao sacro	Tocar ao longo da coluna do pescoço até o sacro
16	Spinal Incurvation	Curvatura espinhal	Curvamento da Coluna	Curvatura espinhal
16	Stroke right then left thoracolumbar paraspinals	Acertar a coluna à direita e depois à esquerda e depois deixá-la reta.	Movimentar paraespinhais toracolombares para a direita e para a esquerda	Tocar paraespinhais toracolombares à direita e à esquerda.
16	Tickle abdomen or foot or tilt in infants with integrated Galant	Fazer cócegas no abdômen ou no pé da criança, com inclinação integrada Galant	Estimula abdomen ou pé, ou inclina a criança com Galant integrado	Fazer cocégas no abdômen ou nos pés ou inclinar a criança com um Galant integrado

Abbreviations: PTV 1, Portuguese Translated Version 1; PTV 2, Portuguese Translated Version 2; STV, synthesis of translated versions.

At the backtranslation stage, the native English-speaking translators presented their version of the scale translated back into English. Minimal discrepancies between the versions revealed synonyms that did not change the understanding of the expressions, were close to the original version, and were considered appropriate after evaluation by the author of the original scale.

The committee of experts analyzed all previous versions and decided to include the term *duração da avaliação* in the header; replace the term *bed surface* with *superfície* ; replace the term *hand grip* with *preensão palmar* (item 3); replace the phrase *allow the infant to derotate* (item 7) with *permita que a criança tente rolar* ; adjust the term *able to get arm of body* to *retirar o braço de próximo do corpo* (item 8); add the term *gentilmente* to the verb *beliscar* (items 10 and 11); replace *head hangs* with *cabeça fica pendente* (item 12); replace *head/neck extension* with *extensão da cabeça/cervical* (item 15); and replace *spinal incurvation* with *flexão lateral da coluna* in item 16. The content of each item of the scale in PTV1 and PTV2 was validated by the committee of experts ( Table 2 ). All agreement rates were > 0.8, and the overall CVI of the instrument was 0.98, calculated as the mean value of the item divided by the number of items.

**Table 2:** Content validity index – committee of experts

Item / experts	1	2	3	4	5	6	7	8	9	10	CVI
1	4	4	4	4	3	4	3	4	4	4	1
2	4	4	4	4	4	4	4	4	4	4	1
3	3	4	4	3	4	3	4	3	3	4	1
4	4	4	4	4	4	3	4	4	4	4	1
5	4	4	3	3	3	4	4	3	3	4	1
6	3	3	3	3	3	3	4	3	3	4	1
7	4	3	4	3	4	3	3	3	3	4	1
8	4	3	4	4	4	4	4	4	3	4	1
9	4	4	3	4	3	3	3	4	4	4	1
10	4	4	4	3	4	3	4	4	4	4	1
11	4	4	4	4	4	4	4	4	3	4	1
12	3	3	2	3	4	3	3	3	2	4	0.8
13	4	4	4	3	4	4	4	4	4	4	1
14	4	4	4	4	4	4	4	4	3	4	1
15	4	4	4	3	3	4	3	3	3	4	1
16	3	4	3	4	2	4	3	3	3	4	0.9

Abbreviation: CVI, content validity index.

In the pretest of the final Brazilian version of the CHOP INTEND, the scale was applied to a convenience sample of 13 patients ( Table 3 ). Five out of 18 patients with SMA type I registered at the service were excluded from the study: 3 could not complete the test under spontaneous breathing, while the behavior of 2 patients was not favorable to the assessment. Patients were predominantly male (76.92%) and had a mean age of 17.23 ± 20.24 months old; the mean assessment time was 47.3 minutes.

**Table 3:** Sample characteristics

Patient	Sex	Number of copies of SMN2	Age (months old)
1	M	2	79
2	F	3	20
3	M	3	17
4	M	2	11
5	M	2	32
6	M	3	19
7	F	2	6
8	M	2	3
9	M	2	9
10	M	2	8
11	M	2	4
12	M	2	10
13	F	2	6

Abbreviations: F, female; M, male; SMN2, survival motor neuron 2.

In the reliability analysis, the scores assigned by the three researchers for each item on the scale are shown in Table 4 . The inter-rater reliability was considered excellent (ICC = 0.998).

**Table 4:** Test of the final Brazilian Portuguese version of the CHOP INTEND scale. Score of the three researchers (A, B, and C) per item for each patient

Patient	1	2	3	4	5	6	7	8	9	10	11	12	13																										
Researchers	A	B	C	A	B	C	A	B	C	A	B	C	A	B	C	A	B	C	A	B	C	A	B	C	A	B	C	A	B	C	A	B	C	A	B	C	A	B	C
Items																																							
1	2	2	2	2	2	2	3	3	3	3	3	3	2	2	2	2	2	2	3	3	3	3	3	3	3	3	3	3	3	3	4	4	4	4	4	4	3	3	3
2	2	2	2	0	0	0	2	2	2	2	2	2	1	1	1	1	1	1	2	2	2	2	2	2	2	2	2	1	1	1	3	3	3	3	3	2	2	2	2
3	1	1	1	0	0	0	2	2	2	4	4	4	0	0	0	0	0	0	2	2	2	3	3	3	4	4	4	3	3	3	4	4	4	4	4	4	4	4	4
4	2	2	2	2	2	2	4	4	4	4	4	4	0	4	4	2	2	2	4	4	4	4	4	4	4	4	4	4	4	4	4	4	4	4	4	4	4	4	4
5	4	4	4	0	0	0	2	2	2	4	4	4	0	0	0	2	2	2	2	2	2	4	4	4	0	0	0	0	0	0	4	4	4	0	0	0	0	0	0
6	1	1	0	0	0	0	1	1	1	2	2	2	1	1	1	0	0	0	2	2	2	1	1	1	2	2	2	1	2	1	2	2	2	2	2	2	2	2	2
7	1	2	2	0	0	0	1	1	1	2	2	2	0	0	0	0	0	0	1	1	1	1	1	1	2	2	2	1	1	1	2	2	2	2	2	2	2	2	2
8	1	1	1	1	1	1	3	3	3	3	3	3	1	1	1	0	0	0	3	3	3	3	3	3	3	3	3	1	1	1	4	4	4	4	4	4	2	2	2
9	1	1	1	0	0	0	1	1	1	3	2	2	0	0	0	0	0	0	1	1	1	1	1	1	1	1	1	1	1	1	2	2	2	4	4	4	2	2	2
10	2	2	2	0	0	0	1	1	1	2	2	2	0	0	0	0	0	0	2	2	2	1	1	1	2	2	2	0	0	0	4	4	4	4	4	4	4	4	4
11	0	2	0	0	0	0	3	3	3	NPT	NPT	NPT	2	2	2	0	0	0	2	2	2	2	2	2	3	3	3	2	2	2	3	3	3	3	3	3	0	1	2
12	0	0	0	0	0	0	0	0	0	3	3	4	0	0	0	0	0	0	2	2	2	0	0	0	2	2	2	0	0	0	2	2	2	2	2	2	2	2	0
13	0	0	0	0	0	0	0	0	0	4	4	4	0	0	0	0	0	0	0	0	0	0	0	0	0	0	0	0	0	0	4	4	4	2	2	2	4	4	4
14	0	0	0	0	0	0	0	0	0	2	2	2	0	0	0	0	0	0	0	0	0	0	0	0	0	0	0	0	0	0	2	2	2	2	2	0	0	0	0
15	0	0	0	0	0	0	0	0	0	0	0	0	0	0	0	0	0	0	0	0	0	0	0	0	0	0	0	0	0	0	0	0	0	0	0	0	0	0	0
16	0	0	0	0	0	0	0	0	0	4	4	4	0	0	0	0	0	0	0	0	0	0	0	0	2	2	2	0	0	0	0	0	0	4	4	4	0	0	0
SCORE	17	20	17	5	5	5	23	23	23	42	41	42	7	11	11	7	7	7	26	26	26	25	25	25	30	30	30	17	18	17	44	44	44	44	44	41	31	32	31

Abbreviations: NPT, Not Possible to Test.

## DISCUSSION

The translation allowed adjustments and substitutions of the terms to guarantee equivalence based on the consensus of researchers, translators, and experts. Content validity showed agreement rates > 0.8, the overall CVI of the instrument was 0.98, and the final Brazilian Portuguese version presented good inter-rater reliability (ICC = 0.998).

The current scenario of proactive care and therapeutic innovations indicates a change in the phenotypes of patients with SMA type I. 27 28 Therefore, the use of standardized measures for assessment is crucial to integrate clinical practice with research. 29 30 Besides, the scales currently available in Brazil to assess infant development require different postures, are not sensitive, specific, or responsive to changes, and may produce a floor effect in patients with SMA type I. 10 15

The CHOP INTEND is a valid instrument used worldwide, which makes its translation into Brazilian Portuguese more convenient than creating a new instrument. 10 15 The recommended requirements were followed to enable a proper and accurate Brazilian version of the CHOP INTEND and guarantee the quality of results. 20

We did not find publications on CHOP INTEND translation and cross-cultural adaptation protocols for other languages/countries. An international multicenter study, about a training protocol for evaluators of clinical trials in countries of Europe, Asia, and the Pacific region, reported that the test materials applied to patients with SMA, including the CHOP INTEND, were only translated into the local language based on the needs of the evaluators. 16

In our translation methodology, according to a formal protocol, the difficulties encountered (for example, idiomatic expressions, cultural variations, and regionalisms) were resolved by consensus between translators, researchers, and experts, who addressed the most appropriate terms in Brazilian Portuguese. We also carefully maintained the aesthetic aspect of the instrument and kept it close to the original document.

Although the convenience sample of 13 patients was small, we considered it adequate for this type of methodological study because SMA is a rare disease with possible respiratory complications, such as recurrent infections requiring invasive mechanical ventilation, and short life expectancy. 31 32

In the development study of CHOP INTEND, the intrarater reliability analysis included only 9 patients and the inter-rater reliability test, considering its possible application in other rare neuromuscular conditions in childhood, involved 10 children with other diseases. 13

In further evaluation for concurrent validation of the scale, 27 patients with SMA from 3 to 260 months old (81% of them < 5 years old) were included through a multicenter study, which correlated CHOP INTEND scores with the time of NIV use. It was shown that patients who were older and required longer ventilation time presented lower scores on the scale. 14 As in this study, our sample was also composed of subjects who did not require invasive ventilation.

Therefore, we believe the Brazilian version of the CHOP INTEND met semantic and technical equivalence criteria with the original version and presented excellent content validity and reliability to support its use in Brazilian patients with SMA type I. ( **Supplementary Material** - https://www.arquivosdeneuropsiquiatria.org/wp-content/uploads/2023/09/ANP-2022.0302-Supplementary-Material.zip ).
